# LZM008, a proposed tocilizumab biosimilar: Pharmacokinetics, safety, and immunogenicity profiles compared with ACTEMRA^®^ in Chinese healthy male subjects

**DOI:** 10.3389/fphar.2023.1111893

**Published:** 2023-04-04

**Authors:** Guoying Cao, Jingjing Wang, Jinjie He, Yingying Hu, Haijing Yang, Linling Que, Xianghong Gu, Jicheng Yu, Xiaojie Wu, Jufang Wu, Wei Fang, Qing He, Jing Zhang

**Affiliations:** ^1^ Phase I Clinical Research Center, Huashan Hospital of Fudan University, Shanghai, China; ^2^ Drug Clinical Trial Institution, The Wuxi People’s Hospital of Nanjing Medical University, Wuxi, Jiangsu, China; ^3^ Livzon Mabpharm Inc., Zhu Hai, Guangdong, China

**Keywords:** biosimilar, tocilizumab, pharmacokinetics, immunogenicity, safety

## Abstract

**Background:** This study aimed to investigate the pharmacokinetics, safety, and immunogenicity of recombinant humanized anti-human IL-6R monoclonal antibody injection, LZM008, and evaluate the pharmacokinetic similarity between LZM008 and tocilizumab (ACTEMRA^®^) in Chinese healthy male subjects.

**Research design and methods:** In this randomized, double-blinded, paralleled, two-center Phase I clinical trial, 96 subjects were randomized with a 1:1 ratio to receive 4 mg/kg intravenous dose of LZM008 or ACTEMRA^®^ and evaluated for 28 days. The pharmacokinetic bioequivalence was assessed by the maximum serum concentration (C_max_), the area under the serum concentration–time curve (AUC) from time 0 to the last detectable drug concentration (AUC_0-t_), and AUC_0-∞_. The statistical analysis was conducted using SAS Enterprise Guide statistical software. Safety was assessed by physical examinations, vital signs, laboratory tests, and electrocardiograms. Anti-drug antibodies (ADAs) were measured by a bridged electrochemiluminescence immunoassay.

**Results:** LZM008 (N = 49) and ACTEMRA^®^ (N = 47) groups showed similar pharmacokinetic properties. After a single intravenous infusion of 4 mg/kg LZM008, the C_max_ and AUC_0-∞_ values of LZM008 reached 87.99 μg/mL and 11,526.70 h*μg/mL, respectively, with T_max_ 1.98 h, and the half-life (t_1/2_) was 83.45 h. The 90% confidence intervals of ratios for C_max_, AUC_0-t_, and AUC_0-∞_ were within the range of 80.00%–125.00%. After infusion, one (2.0%) subject in the LZM008 group and three (6.4%) subjects in the ACTEMRA^®^ group showed positive ADA test results. The incidence of treatment emergent adverse events (TEAEs) was comparable in LZM008 and ACTEMRA^®^ groups (98.0% *versus* 100%), with the decrease in blood fibrinogen and neutrophil counts being the most common TEAEs.

**Conclusion:** The pharmacokinetic characteristics and immunogenicity exhibited by LZM008 were similar to those of the reference product, ACTEMRA^®^. The safety profiles of LZM008 were similar in the two groups with mild–moderate adverse effects.

**Trial Registration:** The trial is registered at www.chinadrugtrials.org.cn (CTR20190889).

## 1 Introduction

Rheumatoid arthritis (RA) is a systemic inflammatory disorder with a prevalence of 0.46% worldwide (Almutairi et al., 2021). The chronic inflammatory state of RA can lead to extra-articular manifestations, such as rheumatoid nodules and vasculitis (Smolen et al., 2016), and to cardiovascular, pulmonary, gastrointestinal, and other diseases (Marcucci et al., 2018), leading to increased morbidity and premature mortality. Tocilizumab, a recombinant humanized IgG1κ monoclonal antibody, is the first therapeutic monoclonal antibody product that targets the interleukin 6 (IL-6) receptor and has been approved to treat RA by reducing acute phase reactants, hepcidin production, B-cell activation, bone resorption, and cartilage transformation, and inhibiting T-lymphocyte differentiation into Th17 cells (Nishimoto and Kishimoto, 2008; Tanaka et al., 2010; Navarro-Millan et al., 2012; Biggioggero et al., 2019). Meanwhile, coronavirus disease 2019 (COVID-19)-related multiple-organ failure and ARDS are mainly caused by a cytokine storm, which are mainly related to inflammatory cytokines, especially to an IL-6 receptor (Ye et al., 2020), and several research studies have been carried out to evaluate the impact of tocilizumab on the mortality rate and mechanical ventilation incidence (Raiteri et al., 2021).

Biosimilars are highly similar to the approved biological drugs with important characteristics, such as biological activity, pharmacokinetics (PK), immunogenicity, and safety (Agency, 2014; Administration, 2015). Unlike generic products of chemically synthesized drugs, biosimilars are highly similar to the reference product, notwithstanding minor differences in clinically inactive components (Felix et al., 2014), and biologics are highly dependent on the manufacturing steps. The same protein made by different methods or in different facilities would have a different efficacy and safety and purity profile (Schellekens, 2009). Therefore, the process of creating biosimilars is challenging, and biosimilar evaluation is necessary (Daller, 2016).

LZM008, a potential biosimilar of tocilizumab (ACTEMRA^®^), is a recombinant humanized anti-human IL-6R monoclonal antibody independently developed by Livzon Mabpharm Inc. (China). Pharmacodynamic studies at molecular and cellular levels have been carried out in the preclinical study, and studies on pharmacokinetics, pharmacodynamics, and immunogenicity have also been carried out in cynomolgus monkeys (unpublished data). The results of preclinical studies have demonstrated high similarities between LZM008 and tocilizumab (unpublished data). Until now, there is no biosimilar for tocilizumab marketed in China, and LZM008 might be an alternative, with the advantage of lower price and higher accessibility.

This Phase I study in Chinese healthy male subjects was conducted to compare the biosimilarity of LZM008 to the reference originator tocilizumab in PK properties, safety, and immunogenicity.

## 2 Subjects and methods

### 2.1 Study design

This was a randomized, double-blinded, single-dose, parallel-group Phase I clinical trial conducted in Huashan Hospital of Fudan University and Wuxi People’s Hospital (China), which was designed to assess the PK bioequivalence of LZM008 to ACTEMRA^®^ as the primary endpoint, and the safety and immunogenicity similarity of two products as the secondary endpoints. The entire study was carried out in compliance with the Declaration of Helsinki principles and the Good Clinical Practices and Provisions for drug registration issued by the National Medical Products Administration. The ethics committee of the Huashan Hospital of Fudan University approved the study protocols and relative documents. All subjects who participated in the study signed informed consent documents before being screened. This trial was registered at www.chinadrugtrials.org.cn (Number: CTR20190889).

Subjects were randomly allocated in a 1:1 ratio to receive a single intravenous infusion of 4 mg/kg LZM008 or ACTEMRA^®^ in 60 min ± 5 min and were discharged from the Phase I clinical research center on day 4 and were required to return to assess safety, PK, and immunogenicity until the end of the study period (day 28). Blood samples for PK assessments were collected at pre-dose (within 2 h), and at 30 min, 1 h (immediately at the end of the infusion), 2 h, 4 h, 8 h, and 24 h (day 2); 48 h (day 3); 96 h (day 4); 168 h (day 7); 240 h (day 10); 336 h (day 14); 408 h (day 17); 504 h (day 21); 576 h (day 24); and 672 h (day 28). Anti-drug antibody (ADA) tests were performed at prespecified visits, including the screen visits on days 14 and 28. ADA-positive samples were further examined for the presence of neutralizing antibodies (NAbs).

### 2.2 Study subjects

Chinese healthy male subjects (age range of 18–45 years) having a body weight larger than 50 kg, with a body mass index (BMI) ranging from 18 to 26 kg/m^2^ (inclusive), were enrolled into this study. Meanwhile, eligible subjects should use appropriate and effective contraceptive measures, such as abstinence, oral contraceptives, intrauterine devices, and condoms combined with a contraceptive diaphragm. In addition, the major exclusion criteria included 1) the history of clinically significant diseases based on their medical history, physical examinations, vital signs, electrocardiograms, laboratory findings, or chest X-ray; 2) subjects who participated in any other clinical trials and donated blood more than 200 mL in the past 3 months; and 3) positive results during an alcohol breath test, drug abuse test, or nicotine test.

### 2.3 Pharmacokinetic evaluations

An intravenous catheter was used for 8 h in each subject from the predose blood collection. Blood samples of 4 mL were collected in a serum separation tube with a coagulant each time and separation gel and kept at room temperature for 30 min ±15 min, and then centrifuged at 1,500 ∼ 2000 g for 10 ∼ 15 min at a temperature of 2 ∼ 8°C. The supernatant serum was separated and stored at −90 ∼ −60°C within 2 h.

Serum concentrations of LZM008 and ACTEMRA^®^ were determined using an enzyme-linked immunosorbent assay (ELISA), which was methodologically validated. The limit of quantification was 75.0 ∼ 2,400 ng/mL, and all serum concentrations lower than 75 ng/mL were recorded as 0 in the calculation of pharmacokinetic parameters. Meanwhile, if the sample concentration is higher than that in HOQ, the sample concentration would be diluted at a minimum of 30 times for detection. The precision values of an inter-batch were ≤ 20%, and the inter-assay accuracies ranged from 3.0% to ∼ 4.0%.

Pharmacokinetic parameters were calculated using a non-compartmental model (WinNonlin version 6.4), which included the maximum serum concentration (C_max_), time to C_max_ (T_max_), the area under the concentration–time curve (AUC) from time 0 to the last detectable drug concentration (AUC_0-t_), the AUC from time 0 to infinity (AUC_0-∞_), clearance (CL), and elimination half-life (T_1/2_).

### 2.4 Immunogenicity evaluations

Blood samples for immunogenicity analysis were collected at the screening day and days 14 and 28 after infusion. The process of immunogenicity samples was the same as compared to PK samples, and the samples were analyzed for the presence of anti-drug antibodies (ADAs) using a bridging electrochemiluminescence immunoassay (bridging-ECLIA), which was methodologically validated. ADA-positive samples were further examined for the presence of neutralizing antibodies (NAbs). Based on the principle of specific combination of antibodies and drugs, the sample is mixed with a capture reagent (Biotin-Drag) and detection reagent (Drug-SULFO-TAG) to form an immune complex, which can be captured and recognized by the MSD Read Buffer Plate, and the signal (ECLU) is read by using an MSD instrument. The signal value is proportional to the content of the ADAs of the sample.

### 2.5 Safety evaluations

Safety was assessed by physical examinations, vital signs, laboratory tests, and electrocardiograms. The type, timing, seriousness, and relatedness of adverse events (AEs) were documented based on the results of vital signs, clinical laboratory data, and other medications used in this study. Laboratory tests were examined at screening and on days 3, 14, and 28. Vital sign examinations were performed at 3 h pre-dose; 2 h, 4 h, and 12 h after infusion on day 1; and on days 2, 3, 4, 7, 10, 14, 17, 21, 24, and 28 after infusion. Electrocardiographic (ECG) screening was performed 3 h pre-dose, 2 h after infusion on day 1, and on days 3 and 28 after infusion. The AEs were recorded and graded according to the *National Cancer Institute Common Terminology Criteria for AEs* (*CTCAE*; V 5.0) and were coded according to the *Medical Dictionary for Regulatory Activities* (*MedDRA*; V 22.0).

### 2.6 Statistical analyses

The sample size was determined according to previous studies on the bioavailability of ACTEMRA^®^. The level of significance (α) has been set as 0.05, according to the Bonferroni adjustment method, and there should be 95% power for each endpoint at the same time. The coefficient of variation (CV) for AUC_0-∞_ was 11%∼24%, and CV of C_max_ was 8%∼20% for ACTEMRA^®^ (ACTEMRA, 2016); the true ratio of AUC_0-t_ between LZM008 and ACTEMRA^®^ groups was 1 ± 0.05. A total of 84 subjects (42 per group) were needed in this trial. Moreover, considering that there may be about 15% of subject dropout rate, 96 healthy subjects should be enrolled for randomization.

After a logarithmic transformation of PK parameters, C_max_, AUC_0–t_, and AUC_0–∞_, the ANOVA model with fixed effects (the group, the center, and the interaction between the center and the group) was used for analysis, and then, the *t*-test was used based on ANOVA analysis results to determine the pharmacokinetic bioequivalence of LZM008 and ACTEMRA^®^. If the 90% CIs were observed to be within 80%–125% for all three PK parameters, bioequivalence was inferred. The safety analysis set included subjects who were administered the study drug. Descriptive statistics for PK parameters and demographic data were calculated. Between-group differences were assessed using the chi-squared test for categorical variables, the *t*-test for normally distributed continuous variables, and the Wilcoxon rank-sum test for non-normally distributed variables. All statistical analyses were performed using SAS version 9.4.

## 3 Results

### 3.1 Subjects

**FIGURE 1:**
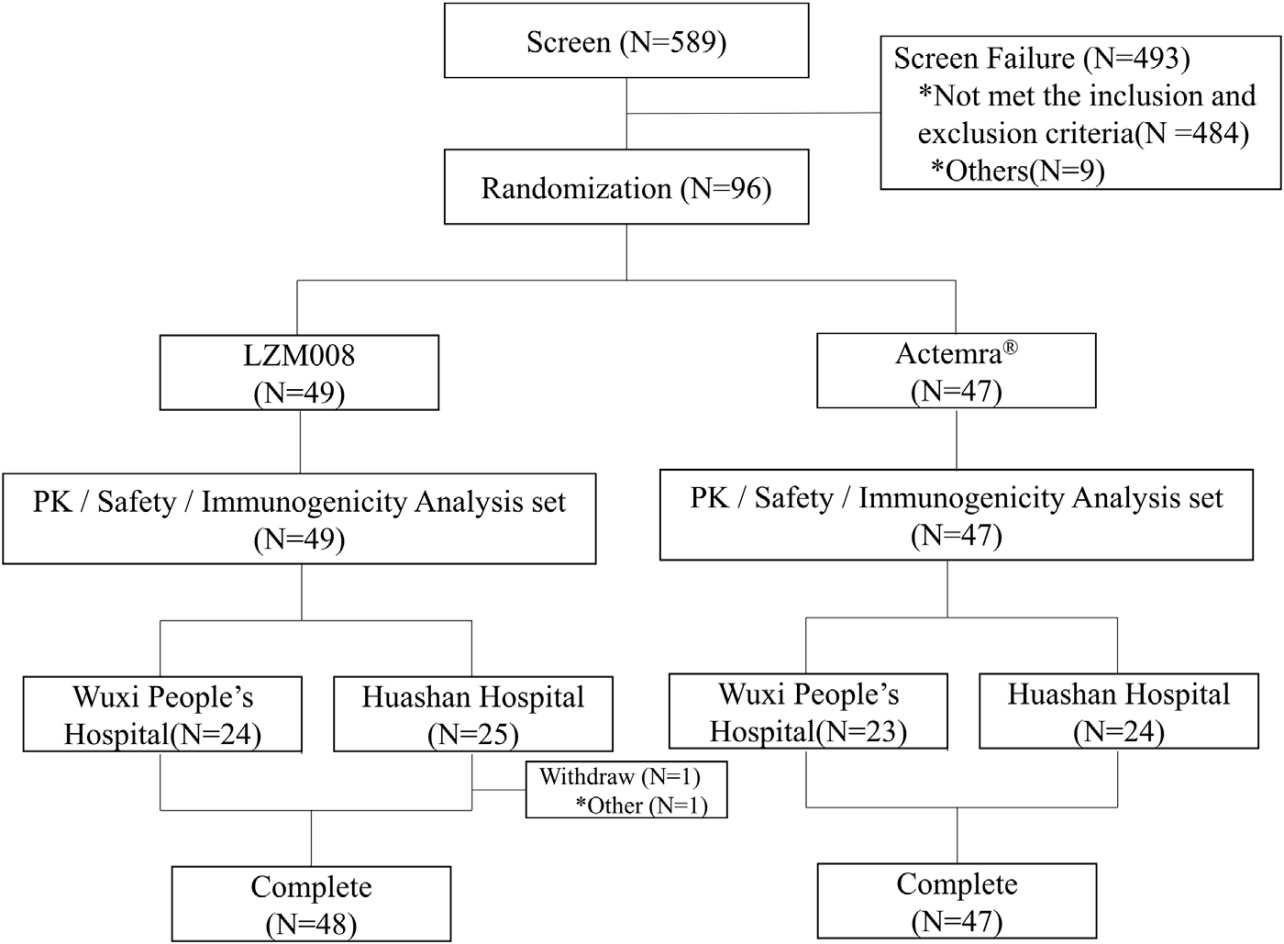
Flowchart of the study.

A total of 589 subjects were screened; 96 eligible male subjects were randomized (49 in the LZM008 group and 47 in the ACTEMRA^®^ group) and received a single intravenous infusion of 4 mg/kg LZM008 or ACTEMRA^®^ (Figure 1). In total, 49 subjects were randomized in the Huashan Hospital of Fudan University, and 47 subjects were randomized in Wuxi People’s Hospital. Although one subject in the LZM008 group withdrew from the clinical trial for personal reasons, with the last PK sample in 504 h and not being included in the bioequivalence set, the remaining 95 subjects completed the trial and entered the full analysis set, PK concentration analysis set, PK parameter analysis set, bioequivalence analysis set, safety analysis set, and immunogenicity analysis set. The two groups were well balanced and comparable in terms of demographics and baseline characteristics (Table 1).

**TABLE 1:** Demographics and characteristics of subjects.

Parameter	LZM008 (N = 49)	ACTEMRA^®^ (N = 47)	Total (N = 96)	*p*-value (LZM008 vs. ACTEMRA^®^)
Age (years)				0.55
Mean ± SD	26.7 ± 4.8	27.3 ± 5.38	27.0 ± 5.1	
Median (range)	26.0 (20.0, 38.0)	26.0 (18.0, 40.0)	26.0 (18.0, 40.0)	
Height (cm)				0.61
Mean ± SD	169.6 ± 6.5	168.9 ± 5.50	169.3 ± 6.0	
Median (range)	168.8 (158.2, 187.1)	169.5 (154.9, 180.0)	169.2 (154.9, 187.1)	
Weight (kg)				0.44
Mean ± SD	64.3 ± 7.7	63.2 ± 6.2	63.7 ± 7.0	
Median (range)	64.9 (50.9–82.0)	63.8 (52.0–79.7)	64.3 (50.9–82.0)	
BMI (kg/m^2^)				0.65
Mean ± SD	22.3 ± 2.1	22.2 ± 1.9	22.3 ± 2.0	
Median (range)	22.4 (18.9, 26.0)	22.2 (18.1, 25.3)	22.3 (18.1, 26.0)	
Ethnicity [N(%)]				
Han nationality	47 (95.9%)	47 (100%)	94 (97.9%)	0.16
Non-Han nationality	2 (4.1%)	0	2 (2.1%)	

BMI, body mass index; SD, standard deviation.

### 3.2 Pharmacokinetic evaluation

**FIGURE 2:**
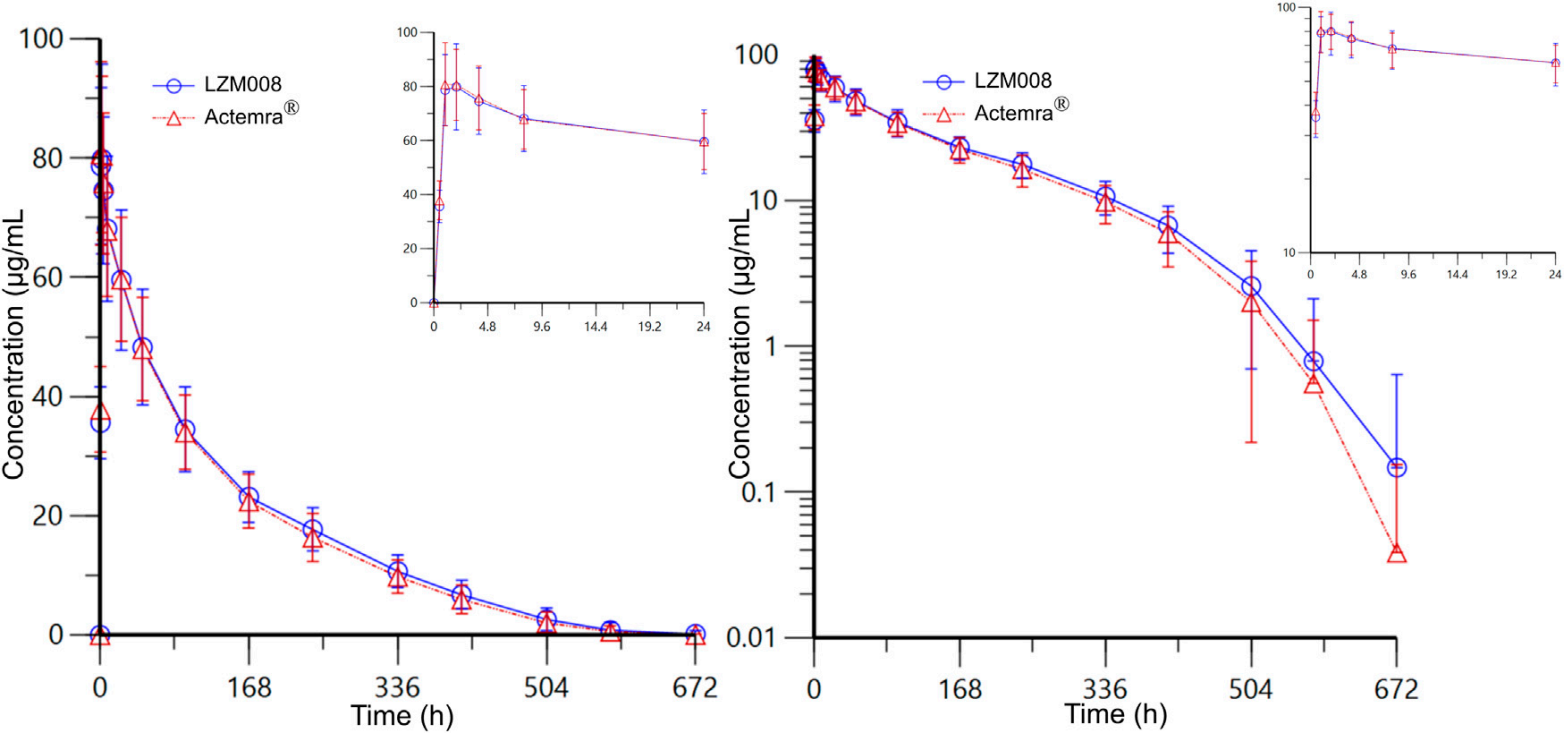
Mean serum concentration–time profiles for LZM008 and ACTEMRA^®^. Note for Figure 2: the error bars represent standard deviation.

The mean serum concentration–time profiles for LZM008 and ACTEMRA^®^ groups were almost overlapped (Figure 2). The AUC_0-t_ and AUC_0-∞_ values of the LZM008 group were slightly higher than those of the ACTEMRA^®^ group, and the CL of the LZM008 group was slightly lower than that of the ACTEMRA^®^ group. The differences in other pharmacokinetic parameters between the LZM008 group and the ACTEMRA^®^ group were all within 10% (Table 2). Meanwhile, the last detectable timepoint of drug concentrations in the LZM008 group was 504 h (12 subjects), 576 h (28 subjects), and 672 h (8 subjects), while in the ACTEMRA^®^ group, the last detectable timepoint of drug concentrations was 408 h (2 subjects), 504 h (23 subjects), 576 h (15 subjects), and 672 h (7 subjects).

**TABLE 2:** Summary of PK parameters of LZM008 and ACTEMRA^®^ after a single infusion of 4 mg/kg.

Parameter	N	LZM008	n	ACTEMRA^®^
C_max_ (μg/mL)	49	87.99 ± 16.31	47	80.68 ± 11.69
AUC_0-t_ (h*μg/mL)	48^a^	11,433.30 ± 2,233.52	47	10,151.3 ± 1780.55
AUC_0-∞_ (h*μg/mL)	48^a^	11,526.70 ± 2,258.29	47	10,234.3 ± 1781.22
T_max_ (h)	49	1.98 (1.00, 8.00)	47	1.98 (1.00, 4.00)
T_1/2_ (h)	48^a^	83.45 ± 27.32	47	77.81 ± 24.05
CL (mL/h/kg)	48^a^	0.36 ± 0.069	47	0.40 ± 0.060
V_d_ (mL/kg)	48^a^	42.65 ± 13.66	47	44.80 ± 15.09

^a^ One subject in the LZM008 group only collected C_max_ and T_max_ due to the missing serum sample from 240 h to 672 h (except 504 h).

All PK parameters were expressed as mean ± SD, except for T_max_, which is expressed as median (minimum and maximum).

After a single intravenous infusion of 4 mg/kg of LZM008, the C_max_ and AUC_0-∞_ values reached 87.99 μg/mL and 11,526.70 h*μg/mL, respectively, with T_max_ 1.98 h, and the half-life (t_1/2_) was 83.45 h. The geometric mean ratios and 90% CI of AUC_0-t_ and C_max_ were 112.06% (105.46% and 119.08%) and 108.38% (102.50% and 114.60%), respectively. In addition, the geometric mean ratio and 90% CI of the secondary endpoint (AUC_0-∞_) were 112.02% (105.44% and 119.02%). The 90% CIs of the GM ratio of all endpoints were still within the range of 80.00%–125.00%. Overall, PK profiles were similar in the two groups and can meet the bioequivalence criterion (Table 3).

**TABLE 3:** Statistical analysis of C_max_, AUC_0-t_, and AUC_0-∞_ of LZM008 and ACTEMRA®.

Parameter	Group	N	GM Mean	95% CI	Ratio [% LZM008/ Actemra^@^]	90% CI
Primary						
C_max_ (μg/mL)	LZM008	49	86.56	82.61, 90.70	108.38	102.50, 114.60
	Actemra^@^	47	79.87	76.15, 83.76		
AUC_0-t_ (h^∗^μg/mL)	LZM008	48^a^	11225.1	10666.5, 11812.9	112.06	105.46, 119.08
	Actemra^@^	47	10016.7	9513.1, 10546.9		
Secondary						
AUC_0-∞_ (h^∗^μg/mL)	LZM008	48^a^	11315.4	10753.5, 11906.6	112.02	105.44, 119.02
	Actemra^@^	47	10101.0	9594.3, 10634.6		

^a^ One subject in LZM008 group only collected C_max_ and T_max_ due to missing plasma sample from 240 h to 672 h (except 504 h).

In addition, the Wilcoxon rank-sum test was used for determining the T_max_ values of the two groups, and the *p*-value was greater than 0.05, and there was no statistical difference in the T_max_ values of LZM008 and ACTEMRA^®^.

Meanwhile, after the natural logarithm transformation of AUC_0-t_, AUC_0-∞_, and C_max_, the ANOVA model with fixed effects (the group, the center, and the interaction between the center and the group) was used for analysis, and then, the *t*-test was used based on ANOVA analysis results to determine the pharmacokinetic bioequivalence of LZM008 and ACTEMRA^®^. The findings of the ANOVA test indicated that there were no significant differences, so the *t*-test was not required. However, in order to maintain consistency with the statistical analysis plan, *t* tests were still carried out. The *p*-values of all fixed effects were larger than 0.05. Therefore, there is no interaction between the center and the experimental grouping, and the effect of the experimental grouping between the two centers remains consistent.

### 3.3 Safety results

The incidence of treatment emergent adverse events was similar in LZM008 and ACTEMRA^®^ groups (98.0% *versus* 100%) (Table 4). In the LZM008 group, 48 (98.0%) subjects had 189 cases of TEAEs, of which 160 cases were related to the investigated drug and 4 (8.2%) subjects had 10 cases of TEAEs of grade III or worse; 182 cases of TEAEs were observed in 47 (100%) subjects in the ACTEMRA^®^ group, of which 161 cases of TEAEs were related to the investigated drug and seven (12.8%) subjects had TEAEs of grade III or worse. There were no SAEs in this study and no TEAEs leading to discontinuation/withdrawal of treatment or death.

**TABLE 4:** Drug-related TEAEs of LZM008 and ACTEMRA®.

AE term	LZM008 (N = 49) n (%)	ACTEMRA^®^ (N = 47) n (%)	Total (N = 96) n (%)
Investigations			
Blood fibrinogen decrease	37 (75.5%)	41 (87.2%)	78 (81.3%)
Neutrophil count decrease	16 (32.7%)	22 (46.8%)	38 (39.6%)
White blood cell count decrease	11 (22.4%)	18 (38.3%)	29 (30.2%)
Neutrophil percentage decrease	7 (14.3%)	7 (14.9%)	14 (14.6%)
Alanine aminotransferase increase	5 (10.2%)	5 (10.6%)	10 (10.4%)
Serum triglyceride increase	5 (10.2%)	6 (12.8%)	11 (11.5%)
Serum bilirubin increase	4 (8.2%)	4 (8.5%)	8 (8.3%)
*Mycobacterium tuberculosis* complex test positive	3 (6.1%)	2 (4.3%)	5 (5.2%)
Aspartate aminotransferase increase	3 (6.1%)	2 (4.3%)	5 (5.2%)
Serum creatinine increase	3 (6.1%)	2 (4.3%)	5 (5.2%)
Conjugated bilirubin increase	1 (2.0%)	4 (8.5%)	5 (5.2%)
Blood uric acid increase	0	3 (6.4%)	3 (3.1%)
Gastrointestinal disorders			
Oral aphthous ulcers	16 (32.7%)	9 (19.1%)	25 (26.0%)
Respiratory, thoracic, and mediastinal disorders			
Oropharyngeal pain	4 (8.2%)	4 (8.5%)	8 (8.3%)
Increased upper airway secretion	3 (6.1%)	0	3 (3.1%)

The incidence of TEAEs of grade III or worse in the trial was similar in LZM008 and ACTEMRA^®^ groups (8.2% vs. 12.8%) (Table 5). One subject in the LZM008 group had four cases of TEAEs of grade IV, which occurred on day 3; we observed a decrease in absolute neutrophils, agranulocytosis, and percentage of neutrophils, and an increase in heterozygous lymphocytes, and 300 μg of granulocyte colony-stimulating factor (G-CSF) was administered for treatment of TEAEs on day 7. All TEAEs of grade IV returned to baseline levels after day 36. Otherwise, five subjects (three in the LZM008 group and two in the ACTEMRA^®^ group) showed *Mycobacterium tuberculosis* complex test positive results at the last follow-up (day 28), while chest CT and symptom assessment results ruled out active tuberculosis infection of five subjects. A follow-up visit is conducted every 3 months for a total of 2 years, and anti-tuberculosis treatment (prophylactic) is considered if necessary.

**TABLE 5:** TEAEs in grade III or higher of LZM008 and ACTEMRA®.

AE term	LZM008 (N = 49) n (%)	ACTEMRA^®^ (N = 47) n (%)	Total (N = 96) n (%)
Investigations			
Neutrophil count decrease	2 (4.1%)	4 (8.5%)	6 (6.3%)
White blood cell count decrease	1 (2.0%)	0	1 (1.0%)
Lymphocyte percentage increase	1 (2.0%)	0	1 (1.0%)
Lymphocyte count decrease	1 (2.0%)	0	1 (1.0%)
Lymphocyte morphology abnormality	1 (2.0%)	0	1 (1.0%)
Serum triglyceride increase	1 (2.0%)	0	1 (1.0%)
Blood fibrinogen decrease	1 (2.0%)	1 (2.1%)	2 (2.1%)
Neutrophil percentage decrease	1 (2.0%)	1 (2.1%)	2 (2.1%)
Blood and lymphatic system disorders			
Agranulocytosis	1 (2.0%)	1 (2.1%)	1 (1.0%)
Gastrointestinal disorders			
Oral aphthous ulcers	0	1 (2.1%)	1 (1.0%)

### 3.4 Immunogenicity results

All randomized subjects showed ADA-negative results at baseline. At the end of the trial (day 28), one (2.0%) subject in the LZM008 group and three (6.4%) subjects in the ACTEMRA^®^ group had ADA-positive test results, with the same titer value of 1:50. Meanwhile, one subject in the ACTEMRA^®^ group was observed to be NAb-positive on day 28, but there were no AEs attributable to the ADA finding for those subjects, and the PK profile of these subjects was similar to that of other subjects with ADA-negative results. Therefore, the incidence of ADA positivity in the LZM008 group was slightly lower than that in the ACTEMRA^®^ group, but it did not show clinically significant differences.

## 4 Discussion

This Phase I clinical trial was designed to evaluate biosimilarity regarding the pharmacokinetic parameters (C_max_, AUC_0-t_, and AUC_0-∞_) and also to compare safety and immunogenicity in Chinese male subjects after a single intravenous injection of 4 mg/kg of either LZM008 or ACTEMRA^®^. The study met its endpoints by showing that the 90% CI of the GM ratio (LZM008/ACTEMRA^®^) for C_max_, AUC_0-t_, and AUC_0-∞_ within the bioequivalence margin (80%–125%). The immunogenicity and safety of LZM008 was also similar to that of ACTEMRA^®^.

The dose selection in this study was based on considerations of sensitive doses, clinically recommended doses, and safety of healthy subjects. The recommended dose of ACTEMRA^®^ in RA patients is 8 mg/kg, but when there is an abnormality in liver function , decreased neutrophil counts , or decreased platelet counts, the dose of ACTEMRA^®^ would be reduced to 4 mg/kg. Meanwhile, the recommended starting dose for RA patients is 4 mg/kg of ACTEMRA^®^ in the FDA instructions for ACTEMRA^®^ (ACTEMRA, 2016). In addition, according to a single-dose escalation clinical study of tocilizumab in healthy subjects (unpublished), the AUC_0-∞_ value of ACTEMRA^®^ increased more than the dose escalation in the range of 2–10 mg/kg compared with the dose range of 10–28 mg/kg, which indicated that 4 mg/kg is more sensitive to concentration changes than 8 mg/kg of tocilizumab in subjects. Therefore, 4 mg/kg was selected as the dose for the similarity research in this study.

Studies have shown that IL-6 is closely related to the fibrinogen gene promoter. Tocilizumab, as an IL-6 receptor inhibitor, may inhibit fibrinogen expression (Zhang et al., 1995). After the administration of 8 mg/kg tocilizumab in patients with rheumatic diseases, the lowest fibrinogen value was observed to be 0.82 g/L (Döndü et al., 2019). In this trial, the lowest value of fibrinogen of subjects was all above 1.0 g/L, and disorders of the coagulation and fibrinolysis system have not been observed. Meanwhile, the common adverse effects of tocilizumab included gastrointestinal disorders, such as oral aphthous ulcers. In this study, the incidence of gastrointestinal disorders in the LZM008 group was slightly higher than that in the ACTEMRA^®^ group, but there was no significant difference after statistical analysis. A total of five subjects showed *Mycobacterium tuberculosis* complex test positive results. Although the tuberculosis (TB) infection rate was found to be less common in patients treated with ACTEMRA® when compared with patients treated with tumor necrosis factor (TNF) inhibitors (Gomez-Reino et al., 2003; Vollenhoven et al., 2009) for ACTEMRA^®^ targeted in IL 6, which showed less effects in anti-tuberculosis infection than that in TNF-α, IL-2, and INF-γ (Sutherland et al., 2009), patients with RA still need to be aware of the risk of TB infection when using LZM008.

The drug structure plays a significant role in the immunogenicity profile of a monoclonal antibody, and it can also lead to other changes in the pharmacokinetic profile. Compared with mouse derived monoclonal antibodies, the immunogenicity of monoclonal antibodies with antibody structures composed of human constant regions and mouse variable regions is significantly reduced (Foote and Hwang, 2005). As LZM008 and tocilizumab are humanized immunoglobulin forms, they are much less immunogenic than the chimeric alternatives (Gabay et al., 2013).

One review of double-blinded controlled studies comparing the efficacy of tocilizumab with other biological agents in RA patients with an inadequate response noted that tocilizumab had similar ACR20 (efficacy evaluation index of the American College of Rheumatology) and ACR50 responses but a superior ACR70 response at 24–30 weeks when compared with adalimumab, infliximab, and etanercept (Bergman et al., 2010). However, this study only demonstrated the consistency of the pharmacokinetic characteristics of LZM008 and ACTEMRA^®^, and the efficacy of LZM008 needs to be verified in a larger sample of RA patients in the future. Meanwhile, we did not include female subjects in this study, while the Phase III clinical trial conducted in patients (CTR20191,204) included female subjects to confirm the pharmacokinetics, safety, and pharmacodynamics of LZM008 in female subjects.

## 5 Conclusion

In this study, after a single intravenous injection of LZM008 (a potential tocilizumab biosimilar) or ACTEMRA^®^ in Chinese healthy male subjects, the pharmacokinetic evaluations (C_max_, AUC_0-t_, and AUC_0-∞_) of two groups demonstrated biosimilarity, with a similar pharmacokinetic profile, safety, and immunogenicity data. LZM008 can further be developed as a potential tocilizumab biosimilar in RA patients.

## Data Availability

The raw data supporting the conclusion of this article will be made available by the authors, without undue reservation.
